# HPV E7-mediated NCAPH ectopic expression regulates the carcinogenesis of cervical carcinoma via PI3K/AKT/SGK pathway

**DOI:** 10.1038/s41419-020-03244-9

**Published:** 2020-12-11

**Authors:** Meng Wang, Xiaowen Qiao, Tamara Cooper, Wei Pan, Liang Liu, John Hayball, Jiaxiang Lin, Xiujie Cui, Yabin Zhou, Shule Zhang, Ying Zou, Ranran Zhang, Xiao Wang

**Affiliations:** 1 Department of Pathology, School of Basic Medical Sciences, Shandong University, Jinan, PR China; 2 Department of Radiation Oncology, Qilu Hospital, Cheeloo College of Medicine, Shandong University, Jinan, PR China; 3 Department of Pathology, Qilu Hospital, Cheeloo College of Medicine, Shandong University, Jinan, PR China; 4 Experimental Therapeutics Laboratory, Clinical and Health Sciences, University of South Australia Cancer Research Institute, Adelaide, SA Australia; 5 Key Laboratory for Experimental Teratology of Chinese Ministry of Education, The Shandong Provincial Key Laboratory of Infection and Immunology, Department of Microbiology, School of Basic Medical Sciences, Shandong University, Jinan, PR China

**Keywords:** Cervical cancer, Medical research

## Abstract

Cervical cancer is one of the most common gynecological tumors in the world, and human papillomavirus (HPV) infection is its causative agent. However, the molecular mechanisms involved in the carcinogenesis of cervical cancer still require clarification. Here we found that knockdown of Non-SMC (Structural Maintenance of Chromosomes) condensin I complex subunit H (NCAPH) gene expression significantly inhibited the proliferation, migration, invasion and epithelial–mesenchymal transition (EMT) of cervical cancer cells in vitro, and restrained xenograft tumor formation in vivo. Intriguingly, HPV E7 could form a positive feedback loop with NCAPH. E7 upregulated NCAPH gene expression via E2F1 which initiated NCAPH transcription by binding to its promoter directly. Silencing of NCAPH reduced E7 transcription via promoting the transition of AP-1 heterodimer from c-Fos/c-Jun to Fra-1/c-Jun. Moreover, the E7-mediated NCAPH overexpression was involved in the activation of the PI3K/AKT/SGK signaling pathway. In vivo, NCAPH expression in cervical cancer tissues was significantly higher than which in normal cervix and high-grade squamous intraepithelial lesion (HSIL) tissues, and its expression was significantly correlated with tumor size, depth of invasion and lymph node metastasis. Patients with high NCAPH expression had a significantly better survival outcomes than those with low-expression, suggesting that NCAPH-induced cell proliferation might sensitize cancer cells to adjuvant therapy. In conclusion, our results revealed the role of NCAPH in the carcinogenesis of cervical cancer in vitro and in vivo. The interaction between E7 and NCAPH expands the mechanism of HPV induced tumorigenesis and that of host genes regulating HPV E7.

## Introduction

Cervical cancer is one of the most common malignant tumors in women all over the world. Every year, 500,000 new cases occur worldwide, and with a mortality rate over 30%^1^. Human papillomavirus (HPV) infection is the primary cause of cervical cancer^2^. The HPV genome is composed of double-stranded DNA, encoding seven early and two late open reading frames. The oncogenic protein E7, one of the early expression proteins, is the key oncoprotein leading to malignant transformation of cervical cells^3^. It can regulate cell cycle, inhibit cell apoptosis, and promote cell invasion and metastasis^3–7^. However, HPV infection alone cannot induce the transformation of host cells, but initiates the process towards oncogenesis^8,9^. Once HPV infects the cervical epithelium, subsequent molecular changes involving a cascade of signaling pathways ultimately promote the development and progression of cervical cancer^10,11^. At present, the detailed molecular mechanism involved in this process require further elucidation.

Human non-SMC (Structural Maintenance of Chromosomes)-condensin I complex subunit H (NCAPH), encoded by the NCAPH gene, also known as BRRN1 (located on chromosome 2q11.2), functions to maintain the stability of condensin protein complexes and ensure the precise separation of sister chromatids during cell mitosis^12–14^. In 2007, Ryu et al. reported for the first time that the expression of NCAPH was significantly increased in advanced malignant melanoma^15^. In recent years, researchers found that the high expression of NCAPH was associated with poor prognosis in patients with non-small cell lung cancer and prostate cancer^16,17^. Downregulation of NCAPH inhibited the proliferation, migration, and invasion of several cancer cells significantly^18–20^. Moreover, NCAPH was involved in the regulation of mature chromosome condensation and DNA damage^21^. These data suggest that NCAPH may be a key carcinogen involved in the development and progression of human malignant tumors. However, up to now, the function of NCAPH in human cancers is largely unknown, and the underlying molecular mechanism has not been reported.

In this study, we found that reduced expression of NCAPH can inhibit the malignant growth and the epithelial–mesenchymal transition (EMT) process of cervical cancer cells in vitro, and impede the formation of tumors in nude mice. We confirmed the relationship between NCAPH expression and clinicopathological parameters and prognosis of cervical cancer patients. More importantly, we first elucidated the regulatory loop between HPV E7 and NCAPH, and revealed the regulatory role of NCAPH on the PI3K/AKT/SGK pathway in cervical cancer.

## Materials and methods

### Ethics statement

Human and animal studies were approved by the ethics committee of Medical School of Shandong University. Written informed consent was obtained from all patients before use of the materials.

### Patient population

Paraffin specimens include 165 cases of invasive cervical squamous cell carcinoma (ICSCC), 34 cases of high-grade squamous intraepithelial lesion (HSIL) and 82 cases of normal cervix collected between March 2005 and March 2013. Fresh specimens include 10 cases of normal cervix and 22 cases of cervical cancer collected from July 2016 to July 2017 at the Department of Pathology, Qilu hospital. Hematoxylin and eosin (HE)-stained sections were reviewed by two experienced pathologists. The diagnoses were made according to the World Health Organization Classification of Tumors. Patient information was obtained from patient medical record room at Qilu Hospital. All patients had no history of other tumors, otherwise they would be excluded from observation.

### Follow-up

Patients were followed-up every 3 months until death or March 2018. Data collected included survival time, disease-free time, and development of metastases. The period between the date of operation and death was recorded as the overall survival (OS) time. The period between the date of surgery and recurrence or death was noted as the disease-free survival (DFS) time. Patients who were alive at the last follow-up were marked as censored observations.

### Bioinformatics analysis of genetic changes and gene expression of NCAPH in cancers

The cBioPortal database (http://www.cbioportal.org/) was used to explore the genetic variation and proteins co-expressed with NCAPH in cervical cancer. The JASPAR database (http://jaspar.genereg.net/) was used to predict the potential transcription factor that binds to the promoter of NCAPH gene. The GEPIA (http://gepia.cancer-pku.cn/) and UALCAN database (http://ualcan.path.uab.edu/) were used to determine the differentially expressed genes between normal cervix and cervical cancer samples and to unravel NCAPH gene expression between different normal and cancer tissues. Gene set enrichment analysis (GSEA) was applied to identify the gene sets that were related to NCAPH expression. Data were downloaded from The Cancer Genome Atlas (TCGA, https://tcga-data.nci. nih.gov/tcga/) database and the Gene Expression Omnibus (GEO, https://www.ncbi.nlm.nih.gov/geo/) database. *p* < 0.05 was considered statistically significant.

### Cell lines and culture

The cervical cancer cell lines HeLa and SiHa were purchased from the American Type Culture Collection (Manassas, VA, USA). The Human retinal pigment epithelial cells (RPE1-pBabe) and HPV16-E7 expressing RPE1 cells (RPE1-16E7) were kindly provided by Prof. Jianxing Chen (the University of Massachusetts Hospital). All the cells were authenticated using STR profile analysis and tested for mycoplasma contamination recently. HeLa and 293T cells were routinely cultured in Dulbecco’s modified Eagle’s medium, SiHa cells were maintained in RPMI-1640 medium, and both RPE1-pBabe cell lines were cultured in a 1:1 blend of DMEM and Ham’s Nutrient Mixture F12 medium (Gibco BRL, Grand Island, NY, USA). All cell lines were cultured at 37 °C with 10% fetal bovine serum (Gibco BRL, Grand Island, NY, USA) and 1% penicillin–streptomycin.

### RNA extraction and real time PCR

Total RNA was extracted from the cells using TRIzol (Ambion, Calsbad, CA, USA) and cDNA synthesized using a first strand cDNA synthesis kit (Toyobo, Osaka, Japan). Real-time PCR was performed using an ABI Prism 7000 Sequence Detection System with SYBR Premix Ex Taq (Takara, Otsu, Japan). The primer sequences for amplification are listed in Supplementary Table S1. GAPDH was used as the internal control.

### Small-interfering RNA (siRNA) and plasmid transfection

Cells were seeded in six-well culture plates at a density of 2 × 10^5^ cells per well, starved in serum-free medium without antibiotics for 24 h, and then transfected with siRNA or plasmid mixed with Lipofectamine 2000 (Invitrogen, Calsbad, CA, USA). The sequences of specific siRNA (GenePharma, Shanghai, China) are listed in Supplementary Table S1. The E2F1 and NCAPH overexpression plasmid, pCMV-E2F1 and pLenti6/V5-DEST-NCAPH, were purchased from the Addgene repository (Sidney St, Suite 550A, Cambridge, MA), and mock vector was used as a control.

### Western blot analysis

Cells were scraped, pelleted and lysed using CelLyticTM MT Cell Lysis Reagent (Sigma, St. Louis, USA) for total protein extraction. Protein concentration was determined using the BCA reagent kit (Beyotime, Shanghai, China). Western blot analysis was performed as previously described^22^. Membranes were probed with primary antibodies listed in Supplementary Table S2. GAPDH was used as the internal control. The protein bands were visualized using an enhanced chemiluminescence kit (Beyotime, Shanghai, China) according to the manufacturer’s instructions.

### Cell proliferation assay

Cell proliferation was assessed using the Cell-Light™ EdU DNA Cell Proliferation kit (Ribobio, Guangzhou, China) and the Cell-counting kit (CCK-8) (Dojindo, Japan). For the EdU assay, cells were seeded into wells of 96-well plates at a density of 1 × 10^4^ cells per well. After EdU labeling, 100 µL of 1× Apollo reaction cocktail was added and cells were stained with 100 µL of Hoechst 33342 and observed under fluorescence microscope (Olympus, Japan). The percentage of EdU positive cells was defined as the proliferation rate. For the CCK-8 assay, cells were seeded into wells of 96-well plates at a density of 3 × 10^3^ cells per well and processed according to the manufacturer’s instruction.

### Colony formation assay

Colony formation was measured as described previously^22^. In brief, cells were seeded into wells of six-well plates at 500 cells per well after 48 h of transfection. After further incubation for 10 days, colonies were stained with Giemsa and counted.

### Cell migration and invasion assays

Migration assays were performed using Transwell inserts (8.0 µm, 24-well format; Corning, NY, U.S.). For the invasion assay, the inserts were pre-coated with Matrigel matrix (BD Science, Sparks, MD, U.S.). Cell migration and invasion assays were conducted as described previously^23^.

### Plasmid construction and Luciferase assay

The 3′-UTR region of the NCAPH gene was synthesized and inserted into the pGL3-Basic vector to construct the plasmid pGL3-NCAPH (Biosune Biotechnology, Shanghai, China). The pGL3-NCAPH, pCMV-E2F1, pGL3-Basic (mock vector), and pCMV (mock vector) plasmids were co-transfected into 293 T cells pairwise. The LCR regions of HPV16/18 with or without the mutation in AP-1 binding sites were synthesized and inserted into the pGL3-Basic vector to construct the plasmids pGL3-HPV16/18 wtLCR (wild type) and pGL3- HPV16/18 mutLCR (mutation type) (Biosune Biotechnology, Shanghai, China). Then the plasmids and mock vector were co-transfected into HeLa and SiHa cells treated with siNCAPH or 293T cells transfected with NCAPH overexpression plasmid separately. Bright-Glo reagent (Bright-Glo™ Luciferase Assay System; Promega, USA) was added 48 h post-transfection and Firefly luciferase activity was measured. Each experiment was performed in triplicate.

### Immunofluorescence staining

Cells were cultured in a six-well plate and transfected with the specific siRNA targeting NCAPH. They were then transferred to the cover slips and fixed with cold acetone. After blocking with goat serum, cells were incubated with mouse anti-human primary antibody against Vimentin (1:50) (Proteintech, Wuhan, China) overnight at 4 °C. After washing with phosphate-buffered saline, the slides were stained with tetraethyl rhodamine isothiocyanate-labeled goat anti-mouse secondary antibody and counterstained with 4′,6-diamidino-2-phenylindole for imaging of the nuclei.

### Immunohistochemistry staining

The immunohistochemistry staining was performed as described previously^22^. The staining results were judged by two pathologists in a single-blinded manner and graded as follows: 0 (no staining); 1 (light yellow); 2 (yellow brown), and 3 (strong brown). The positive proportion of tumor cells was evaluated as follows: 0 (<5% positive cells); 1 (5–25% positive cells); 2 (26–50% positive cells); 3 (51–75% positive cells), and 4 (>75% positive cells). The staining index (SI) was calculated as the score of the staining intensity multiplied by the proportion of positive cells. SI value was graded as 0, (−); ≤4, (+); 5–8, (++); and ≥9, (+++). Specimens scored (−) to (+) were considered negative, and others were positive. If the staining interpretation differed between the 2 investigators, the data for the slide was discarded.

### Nude mice tumor formation assay

Lentivirus containing pGV248-NCAPH shRNA and pGV248 control vector (carrying GFP protein) was synthesized by Shanghai Genechem Co. Ltd. HeLa and SiHa cells were infected with the virus according to the manufacturers’ instructions in the presence of 1 μg/ml puromycin for 6 weeks. The interference efficacy of NCAPH expression was confirmed by Western Blot analysis. The 4-week-old male NOD/SCID mice were randomized into two groups with comparable body weight. 1 × 10^7^ HeLa cells stably interfering with NCAPH and control cells were then injected subcutaneously into the left axillary fossa of the mice. Tumor growth was monitored for 4 weeks in a single-blinded manner. Tumor volume was determined with the formula: Volume = (width)^2^ × length/2. The hematoxylin and eosin (HE) sections of the tumor mass were observed. Non-invasion of the tumor was defined as intact fibrotic capsule and no adjacent stroma invasion. Tumor invasion was recorded as incomplete encapsulation, muscle or vascular invasion.

### Statistical analysis

SPSS 17.0 software (SPSS Inc., Chicago, IL) was used for statistical analysis. The correlation between NCAPH expression and clinicopathologic characteristics were assessed by Chi-square and Spearman correlation test. The difference of NCAPH expression between normal cervix, HSIL, and cervical cancer was evaluated by Chi-square test. Patients’ survival was analyzed by the Kaplan–Meier method and compared using the log-rank test. Analysis of cell biological behaviors with siRNA treatment involved Student’s *t* test. Data are expressed as mean ± SD. *p* < 0.05 was considered statistically significant.

## Results

### NCAPH is overexpressed in cervical cancer and different solid tumors in vivo

**Fig. 1:**
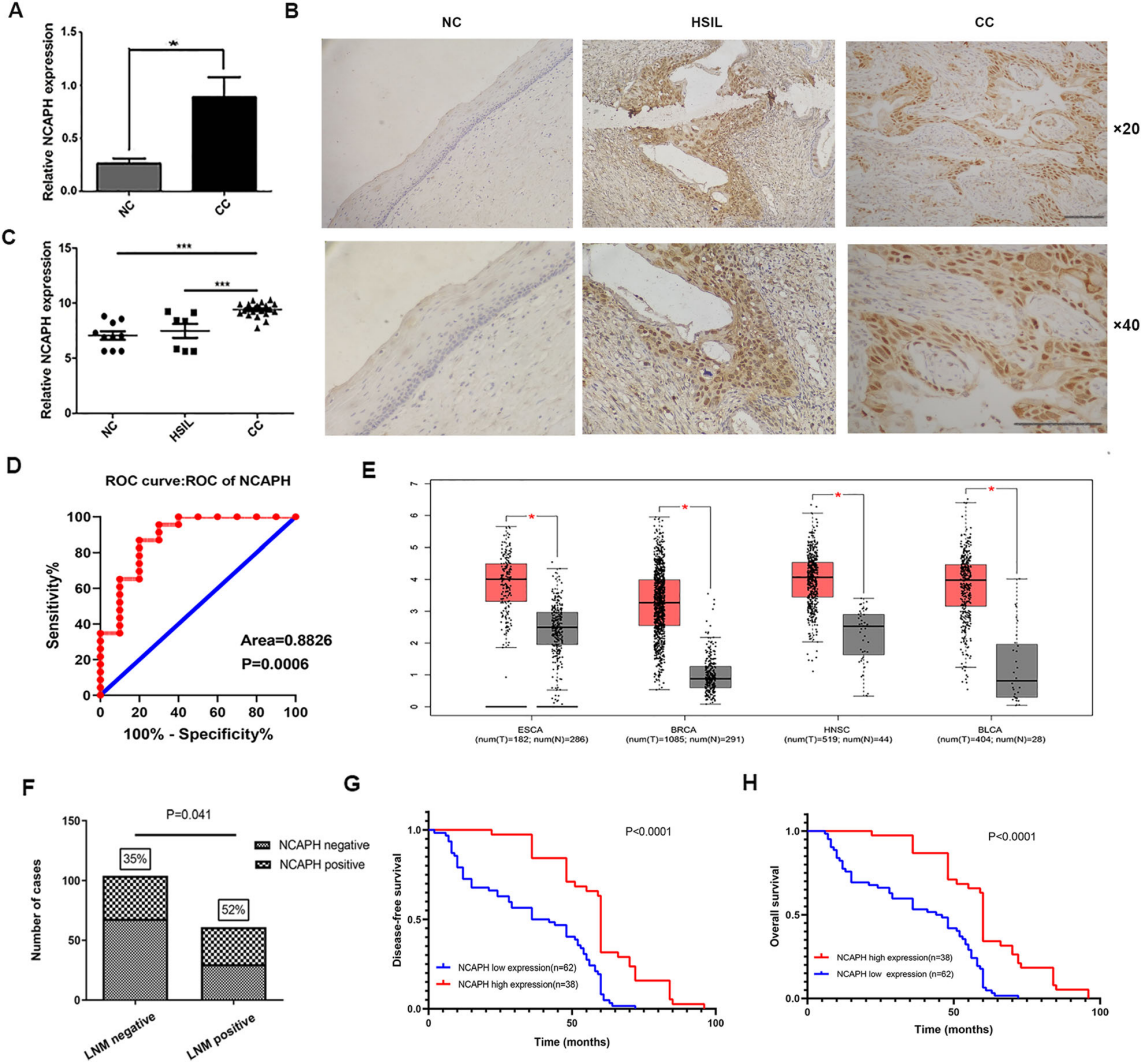
The expression of NCAPH in cervical cancer and its association with patients’ prognosis. **A** The mRNA level of NCAPH in cervical cancer (CC) (*n* = 22) and normal cervix (NC) (*n* = 10) analyzed by real-time PCR. Data are presented as mean ± SD (*n* = 3). **B**, **C)** Immunohistochemical staining of NCAPH in normal cervix (NC) (*n* = 82), high-grade squamous intraepithelial lesion (HSIL) (*n* = 34) and invasive cervical cancer (ICC) (*n* = 165). Scale bars, 100 μm. **D** ROC curves showed the clear separation between normal cervix and cervical cancer tissues, with an area of 0.8826 (*p* = 0.0006). **E** NCAPH expression in several solid tumors analyzed by GEPIA data base. Normal, normal tissues; ESCA, esophageal carcinoma; BRCA, breast invasive adenocarcinoma; HNSC, head and neck squamous cell carcinoma and BLCA, bladder urothelial carcinoma. **F** Compared with LNM-negative cervical cancer (*n* = 104), NCAPH displayed higher expression rate in LNM-positive tissues (*n* = 61) (*p* = 0.0041). Kaplan–Meier curves for survival of 100 patients with cervical carcinoma. Culminative disease-free survival (**G**) and overall survival (**H**).

To explore the role of NCAPH in cervical cancer in vivo, we first determined the NCAPH mRNA level in 10 cases of normal cervical tissues and 22 cases of invasive cervical squamous cell carcinoma (ICSCC) tissues by real-time quantitative PCR. The mRNA level of NCAPH in cervical cancer tissues was significantly higher than that in normal cervical tissues (*p* < 0.05) (Fig. 1A). The results of immunohistochemistry staining showed that NCAPH was expressed mainly in the nucleus but also in the cytoplasm (Fig. 1B). NCAPH was overexpressed in 12.2% of normal cervical squamous epithelium (10/82), 14.7% of high-grade squamous intraepithelial lesion (HSIL) (5/34) and 40.6% of ICSCC (67/165) (Table 1). The expression of NCAPH in cervical cancer was significantly higher than in normal cervix (*p* < 0.001) and HSIL (*p* = 0.004) separately (Table 1; Fig. 1C). Although NCAPH over expression in HSIL was more frequently higher than that in normal cervix, the difference was not statistically significant (*p*å 0.05) (Table 1). The result was further confirmed by the data from GEO database (Supplementary Fig. S1A–C). Moreover, NCAPH was undetectable in 52 cases of normal cervical columnar epithelium and 40 cases of cervical adenocarcinoma (data not shown). Receiver operating characteristic curves demonstrated that NCAPH expression could clearly separate normal cervix and cervical cancer cases, with the area under curve of 0.8826 (Fig. 1D, *p* = 0.0006).

**Table 1:** Expression of NCAPH in cervical tissue samples.

		NCAPH			
Categories	*n*	Negative	Positive	*P value*	*χ*2
normal	82	72	10	0.714	0.135
HSIL	34	29	5		
normal	82	72	10	**0.000**	20.608
ICSCC	165	98	67		
HSIL	34	29	5	**0.004**	8.19
ICSCC	165	98	67		

*normal* normal cervical squamous epithelium, *HSIL* high-grade squamous intraepithelial lesion, *ICSCC* invasive cervical squamous cell carcinoma.

*p* < 0.05 was considered significant (indicated in bold).

To further confirm the important role of NCAPH in cancer, we analyzed the expression of NCAPH in various cancers. The results in GEPIA database showed that the expression of NCAPH was significantly higher in esophageal carcinoma, breast invasive adenocarcinoma, head and neck squamous cell carcinoma, and bladder urothelial carcinoma than that in normal tissues (Fig. 1E). Similar results were obtained by analysis of the data from UALCAN database (Supplementary Fig. S1D).

### The correlation of NCAPH with the clinicopathological parameters and patients’ outcome

We next investigated the relationship between the expression of NCAPH and the clinicopathological parameters of cervical cancer patients. Results showed that the expression of NCAPH was significantly associated with tumor size (*p* = 0.032), depth of invasion (*p* = 0.029) and lymph node metastasis (*p* = 0.041), but not related to patients’ age, FIGO staging, tumor differentiation, or distant metastasis (Table 2), (Fig. 1F) (all *p* values > 0.05).

**Table 2:** Clinicopathological characteristics of patients with cervical cancer and NCAPH status.

		NCAPH			
Categories	N	Negative	Positive	*P value*	*χ*2
Age (y)					
≤40	53	35	18	0.232	1.429
>40	112	63	49		
FIGO stage					
I	152	91	61	0.671	0.180
II & III	13	7	6		
Differentiation grade					
Poor	92	51	41	0.245	1.352
Well to moderate	73	47	26		
Distant metastasis					
No	162	96	66	0.796	0.067
Yes	3	2	1		
Tumor size					
≤4 cm	132	73	59	**0.032**	4.580
>4 cm	33	25	8		
LN metastasis					
No	104	68	36	**0.041**	4.186
Yes	61	30	31		
The depth of invasion					
≤1/2	39	29	10	**0.029**	4.742
>1/2	126	69	57		

*FIGO* International Federation of Gynecology and Obstetrics, *LN* lymph node.

*p* < 0.05 was considered significant (indicated in bold).

Furthermore, we assessed the correlation of NCAPH expression with the patients’ prognosis using Kaplan–Meier survival analysis. Of 165 patients, 100 patients were successfully followed up. These 100 patients were divided into two groups: NCAPH-positive (38 cases) and NCAPH-negative group (62 cases). The disease-free survival (DFS) rate and overall survival (OS) rate were 80.6 % (average DFS time: 59.9 months, 95% CI 53.3–66.6 months) and 82.3 % (average OS time: 62.7 months, 95% CI 57.1–68.2 months) in NCAPH- negative group, respectively. The DFS rate and OS rate were 84.2 % (average DFS time: 86.1 months, 95% CI 79.1–93.0 months) and 86.8% (average OS time: 90.0 months, 95% CI 83.9–96.1 months) in NCAPH-positive group, respectively. The log-rank test revealed that the difference of survival curves between the two groups was statistically significant (*p* <0.05) (Fig. 1G, H) demonstrating that patients with higher NCAPH expression tend to have better prognosis.

### Genetic alterations of NCAPH gene in cervical cancer

We used cBioPortal to study the genetic changes of NCAPH gene in all the two cervical cancer studies. In 607 cases of cervical cancer, one case had NCAPH amplification, three cases had missense mutation (Supplementary Fig. S2). The low rate of genetic alteration indicates that the over-expression of NCAPH in cervical cancer is not mainly induced by NCAPH genetic changes.

### Reduced NCAPH expression effectively inhibits the proliferation and colony formation of cervical cancer cells

**Fig. 2:**
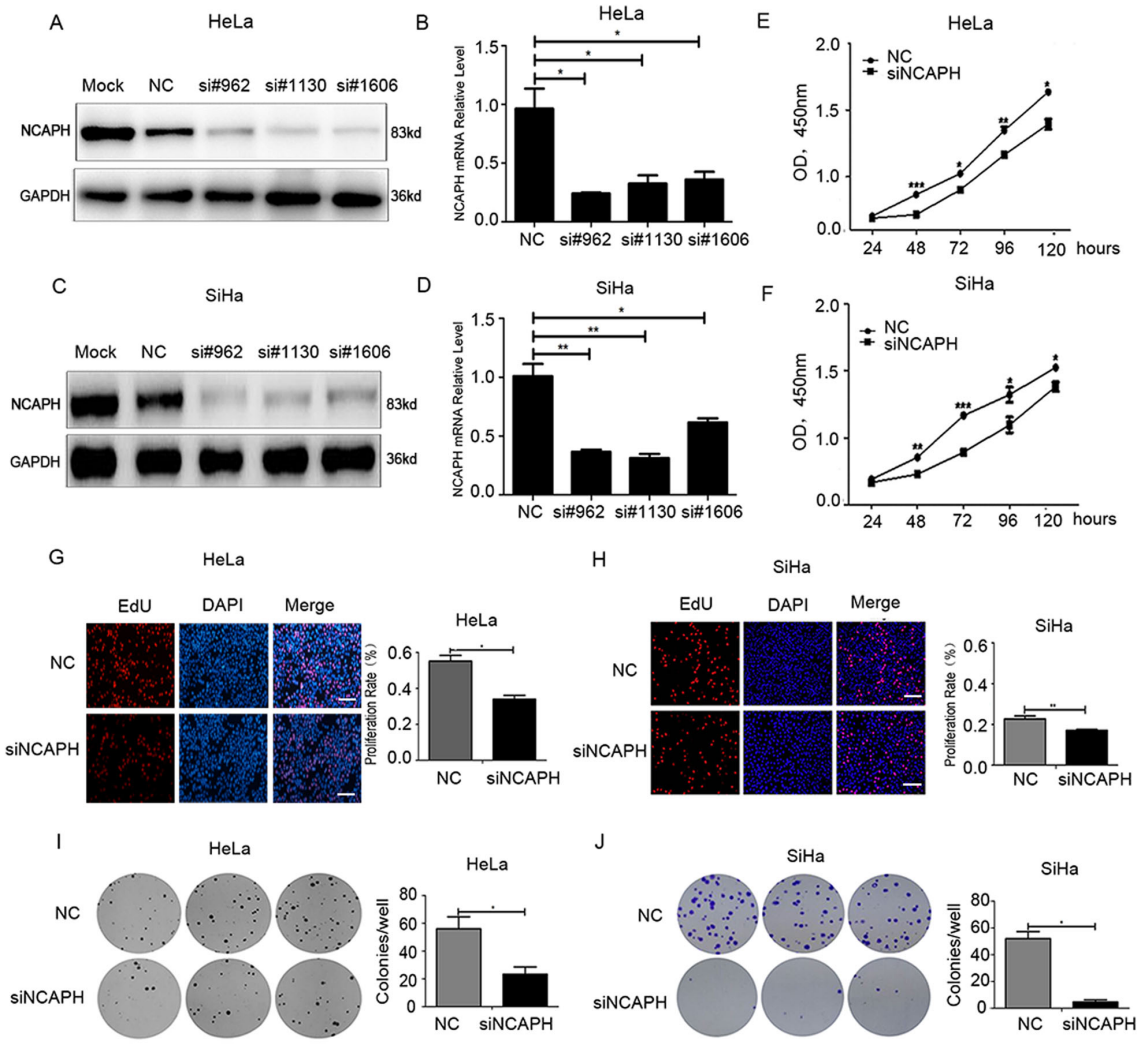
Knockdown of NCAPH expression attenuates the proliferation and colony formation of HeLa and SiHa cells. **A**–**D** The interference efficiency of three NCAPH siRNAs was assessed by Western blot analysis and real-time quantitative PCR. **E**–**H** CCK-8 and EdU assays showed that the proliferation ability of cervical cancer cells decreased significantly when transfected with NCAPH siRNA. Scale bars, 100 μm. **I**, **J** Colony formation assay showed that the capacity of the cells to form colonies was significantly reduced with the knock-down of NCAPH. NC, cells treated with negative control siRNA; siNCAPH, cells treated with siRNA targeting NCAPH gene. Results shown are representative of triplicate experiments. Data are presented as mean ± SD (*n* = 3). * represents *p* < 0.05, ** represents *p* < 0.01, *** represents *p* < 0.001.

To evaluate the biological function of NCAPH in cervical cancer, we designed three pairs of specific siRNAs targeting NCAPH gene, and assessed their interference efficiency in cervical cancer cell lines HeLa and SiHa. Real-time quantitative PCR and Western blot analysis verified that all three siRNAs significantly inhibited the expression of NCAPH in the cells (Fig. 2A–D). NCAPH siRNA #962 was chosen for subsequent experiments. CCK-8 and Edu assay revealed that when transfected with NCAPH siRNA, the proliferation ability of cervical cancer cells was reduced significantly compared with those transfected with NC siRNA (Fig. 2E–H) (all *p* values < 0.05). Consistent with it, NCAPH was significantly co-expressed with PCNA (a specific marker for proliferation ability) in 308 cases of cervical cancer patients from the cBioportal database (Supplementary Fig. S3). Moreover, the colony formation assay showed that once the expression of NCAPH was knocked down, the capacity of HeLa and SiHa cells to form colonies was significantly decreased compared with those transfected with NC siRNA (Fig. 2I, J) (all *p* values < 0.05).

### Reduced NCAPH expression significantly inhibits cell migration, invasion, and EMT process in cervical cancer cells

**Fig. 3:**
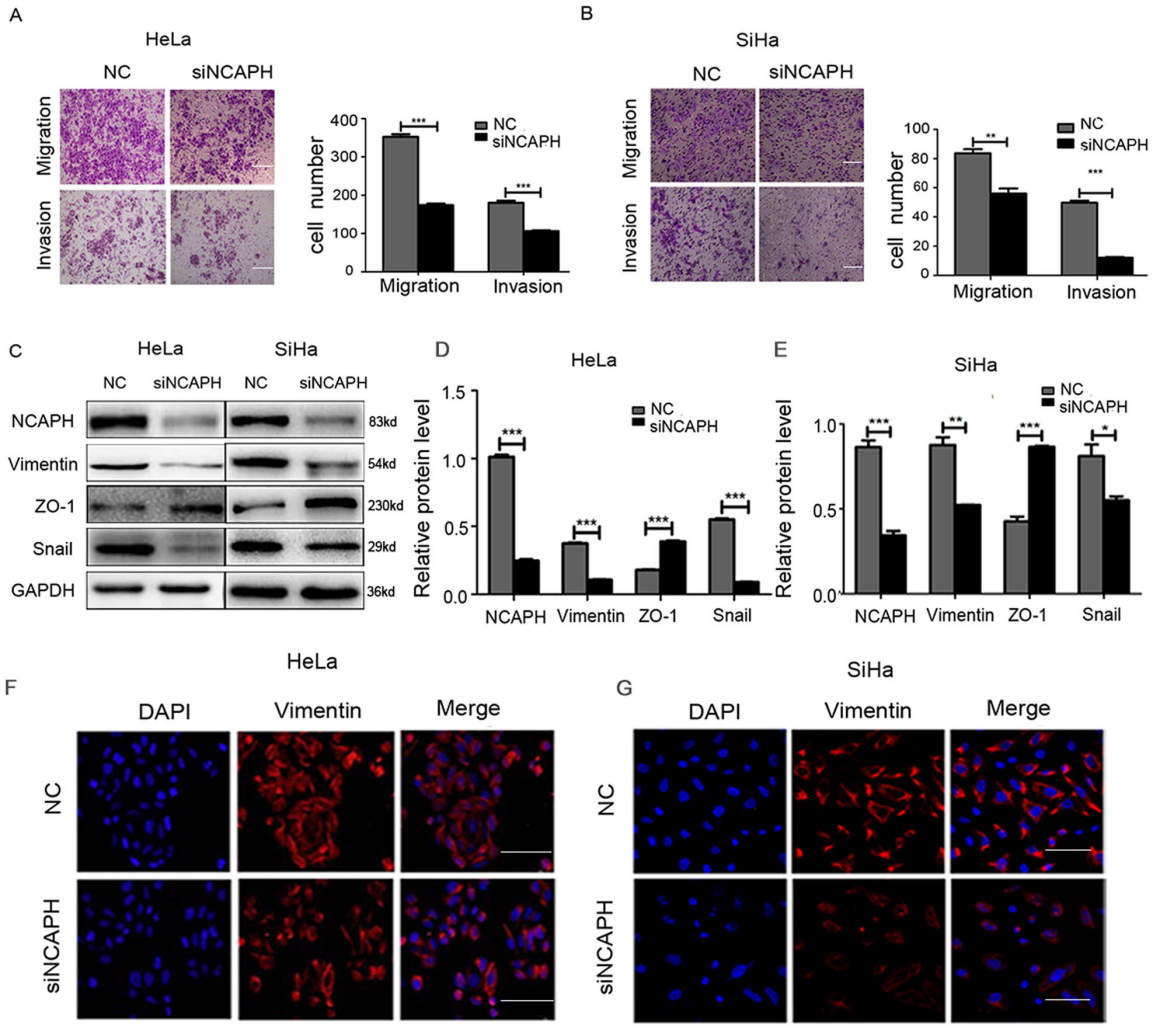
Silencing NCAPH expression reduces cell migration, invasion and EMT process in HeLa and SiHa cells. **A**, **B** Transwell assay results showed that the inhibition of NCAPH expression weakened the migration and invasion capability of HeLa and SiHa cells. Scale bars, 100 μm. **C**–**E** Western Blot analysis showed that the knock-down of NCAPH decreased the protein level of the mesenchymal markers (Vimentin and Snail) while increased that of the epithelial marker (ZO-1). **F**, **G** Immunofluorescent staining confirmed the effects of NCAPH on the expression of Vimentin. NC, cells treated with negative control siRNA; siNCAPH, cells treated with siRNA targeting NCAPH gene. Scale bars, 100μm. Results shown are representative of triplicate experiments. Data are presented as mean ± SD (*n* = 3). * represents *p* < 0.05, ** represents *p* < 0.01, *** represents *p* < 0.001.

To determine the effects of NCAPH on the migration and invasion of cervical cancer cells, we performed transwell assays. Results showed that the number of migrating and invasive cells decreased significantly after reducing the expression of NCAPH in HeLa and SiHa cells (Fig. 3A, B; all *p* values < 0.01). This indicates that the inhibition of NCAPH expression weakens the migration and invasion capability of HeLa and SiHa cells.

To elucidate whether NCAPH influences the migration and invasion of cancer cells via the regulation of the EMT process, we tested the effects of NCAPH on the expression levels of EMT related proteins in HeLa and SiHa cells by Western Blot analysis and immunofluorescent staining. Results showed that when the cells were transfected with NCAPH siRNA, the protein levels of the mesenchymal markers, such as Vimentin and Snail, decreased significantly compared with those transfected with NC siRNA (Fig. 3C–E). In contrast, the interference of NCAPH expression significantly increased the expression level of the epithelial marker ZO-1 (Fig. 3C–E). Similarly, the fluorescence signal of Vimentin decreased significantly when the expression of NCAPH was knocked down in HeLa and SiHa cells (Fig. 3F, G). This suggests that NCAPH promotes the epithelial mesenchymal transition in cervical cancer cells.

### Silencing NCAPH expression dramatically impairs the tumor growth, invasion, and EMT process in nude mice

**Fig. 4:**
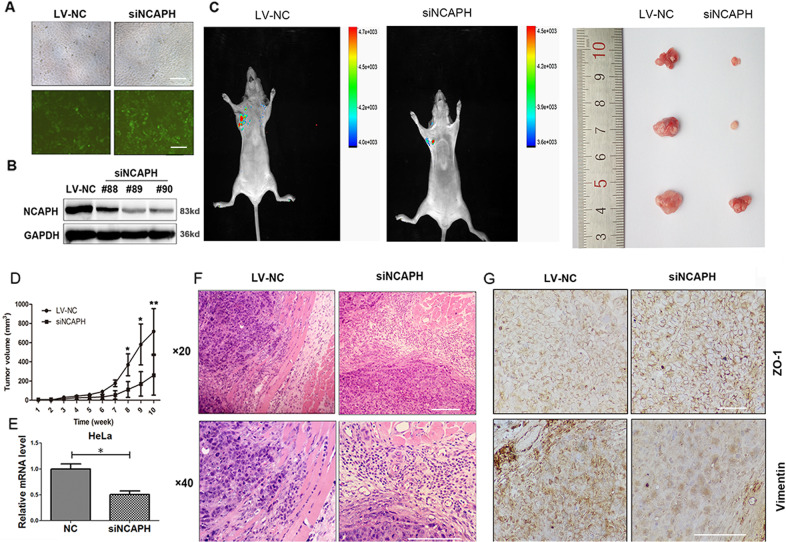
Knockdown of NCAPH expression dramatically impaired the tumor growth, invasion and EMT process in nude mice. **A** Fluorescence microscopy of successful infection of HeLa cells with lentivirus containing pGV248-NCAPH shRNA (siNCAPH) and pGV248 control vector (LV-NC). Scale bars, 100 μm. **B** Western blot analysis confirmed the knockdown of NCAPH in HeLa cells after 6-weeks of selection using puromycin. **C**, **D** The selected HeLa cells were injected into the axillary fossa of the mice. Ten weeks after injection, GFP expressing tumors were imaged in live mice. Then mice were humanely killed, and tumor volumes were calculated (six mice in each group). Representative images are shown. **E** The expression of NCAPH in tumors was determined by real-time PCR. **F** HE staining showed that tumors in the LV-NC group exhibited obvious muscle invasion, but those in the siNCAPH group were well-encapsulated. Scale bars, 100 μm. **G** Immunohistochemical staining showed that ZO-1 protein signal was enhanced while vimentin was weakened after knockdown of NCAPH expression in mice tumors. Scale bars, 100 μm. Data are presented as mean ± SD (*n* = 3). * represents *p* < 0.05, ** represents *p* < 0.01.

HeLa cells were infected with lentivirus containing pGV248-NCAPH shRNA and pGV248 control vector separately. Fluorescence microscopy confirmed that 60% of the tumor cells were successfully infected by the virus (Fig. 4A). Western blot analysis demonstrated that compared with the NC group, NCAPH protein level was dramatically decreased in HeLa cells transfected with pGV248-NCAPH shRNA after 6-weeks of selection using puromycin (Fig. 4B). The selected cells were cultured and injected into the mice for the evaluation of NCAPH function in vivo. Results showed that compared with the NC group, the tumor volume was significantly smaller in mice carrying the tumor cells transfected with pGV248-NCAPH shRNA (Fig. 4C–E). In addition, tumors derived from control vector exhibited obvious muscle invasion indicating strong invasion ability, while tumors established from pGV248-NCAPH shRNA had an intact fibrotic capsule and less adjacent stroma invasion (Fig. 4F). This indicates that knockdown of NCAPH reduces the tumor formation ability of HeLa cells in vivo.

To further confirm the regulatory role of NCAPH in the EMT process in vivo, we performed immunohistochemistry staining in the mouse graft. The results showed that compared with the control group, the signal of ZO-1 protein was enhanced in mice tumors induced by NCAPH knocking down HeLa cells, while the signal of vimentin was significantly weakened (Fig. 4G) (*p* < 0.05). Thus, both in vitro and in vivo results showed that NCAPH plays a vital role in the regulation of EMT processes in cervical cancer.

### HPV E7 initiates the transcription of NCAPH via the transcription factor E2F1 that binds to the promoter region of NCAPH

**Fig. 5:**
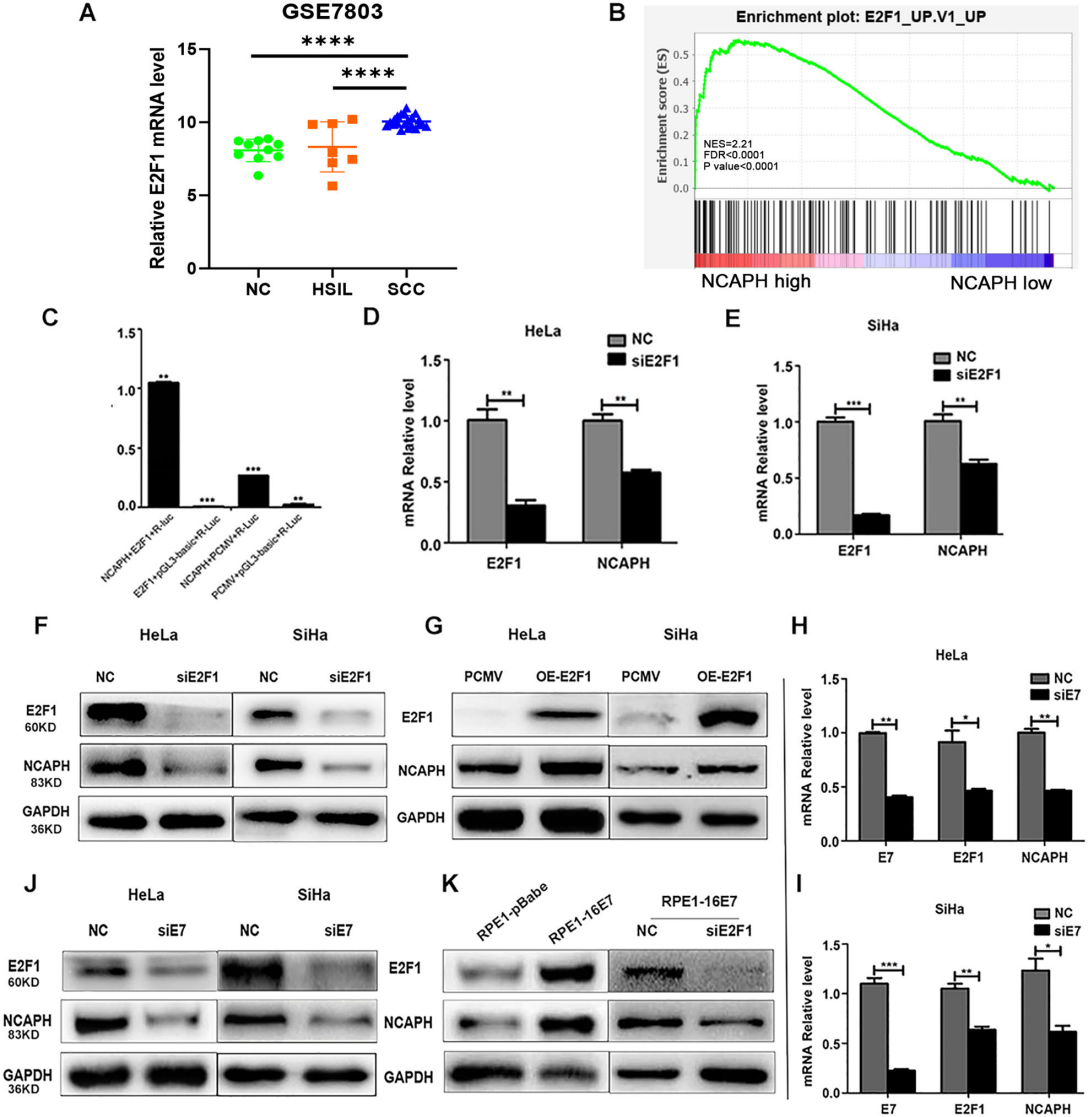
HPV E7 upregulates the gene expression of NCAPH through transcription factor E2F1. **A** The expression of E2F1 in normal cervix (NC) (*n* = 10), HSIL (*n* = 7) and squamous cell carcinoma (SCC) (*n* = 21) based on the data from GEO database (GSE7803). **B** GSEA analysis based on GSE7803 revealed the enrichment of E2F1 with high NCAPH expression. **C** The luciferase assay showed that the luciferase activity increased dramatically when E2F1 overexpression plasmid (pCMV-E2F1) was co-transfected with 3’-UTR regions of NCAPH gene (pGL3-NCAPH) into 293T cells. E2F1, pCMV-E2F1; NCAPH, pGL3-NCAPH; pGL3-Basic, mock vector; pCMV, mock vector. **D**, **E**, **F** Real-time PCR and Western Blot analysis showed that mRNA and protein levels of NCAPH decreased with the knock-down of E2F1 in HeLa and SiHa cells. NC, cells treated with negative control siRNA; siE2F1, cells treated with siRNA targeting E2F1 gene. **G** Overexpression of E2F1 significantly increased the protein level of NCAPH in HeLa and SiHa cells. pCMV, cells transfected with pCMV mock vector; OE-E2F1, cells transfected with pCMV-E2F1 plasmid. **H**, **I**, **J** Real-time PCR and Western Blot analysis showed that E2F1 and NCAPH expression decreased with the knock-down of E7 in HeLa and SiHa cells. NC, cells treated with negative control siRNA; siE7, cells treated with siRNA targeting E7 gene. **K** Western Blot analysis showed that the over-expression of E7 in RPE1-16E7 cells increased the expression of E2F1 and NCAPH significantly. With the interference of E2F1 expression, NCAPH level in RPE1-16E7 cells decreased dramatically. Data are presented as mean ± SD (*n* = 3). * represents *p* < 0.05, ** represents *p* < 0.01, *** represents *p* < 0.001, **** represents *p* < 0.0001.

By analyzing the data from GEO database (GSE7803), we found that E2F1 expression was significantly increased from normal cervix to cervical cancer and from high-grade intraepithelial lesion (HSIL) to cervical cancer (Fig. 5A). Moreover, GSEA analysis revealed that E2F1 was positively correlated with NCAPH expression (Fig. 5B). JASPAR database showed that E2F1 was a potential transcription factor that binds to the promoter of NCAPH gene (data not shown). To verify the predicted result, we performed the luciferase assay. Results showed that the luciferase activity increased dramatically when the pCMV-E2F1 plasmid was co-transfected with the pGL3-NCAPH plasmid indicating that E2F1 could bind to the promoter region of the NCAPH gene (Fig. 5C) (*p* < 0.01). To verify whether E2F1 could initiate the transcription of NCAPH, we transfected HeLa and SiHa cells with E2F1 siRNA and pCMV-E2F1 plasmid separately. Real-time PCR and Western blot analysis revealed that mRNA and protein levels of NCAPH significantly decreased with the knockdown of E2F1 or increased with the over-expression of E2F1 in cervical cancer cells (Fig. 5D–G) (all the *p* values < 0.05). Taken together, these results suggested that the transcription factor E2F1 could bind to the promoter region of NCAPH, thereby activating the transcription and subsequent translation of NCAPH gene.

In view of the strong association between HPV E7 and E2F1, we hypothesized that HPV E7 might regulate the expression of NCAPH via E2F1. To test the hypothesis, we transfected specific E7 siRNA into HeLa and SiHa cells. Results showed that the mRNA and protein levels of E2F1 and NCAPH reduced simultaneously with decreased HPV E7 gene expression (Fig. 5H–J) (all the *p* values < 0.05). We next compared the E2F1 and NCAPH expression levels in RPE1-pBabe cells, RPE1–16E7 cells. Western blot analysis demonstrated that the protein levels of E2F1 and NCAPH were significantly higher in RPE1-16E7 cells compared with RPE1-pBabe cells. Moreover, when RPE1-16E7 cells were transfected with E2F1 siRNA, the elevated NCAPH expression in RPE1-16E7 cells decreased dramatically (Fig. 5K). The data indicated that HPV E7 could up-regulate NCAPH gene expression via E2F1 at the transcriptional level.

### Silencing NCAPH downregulates HPV E7 transcription by conversing AP-1 heterodimer from c-Fos/c-Jun to Fra-1/c-Jun

**Fig. 6:**
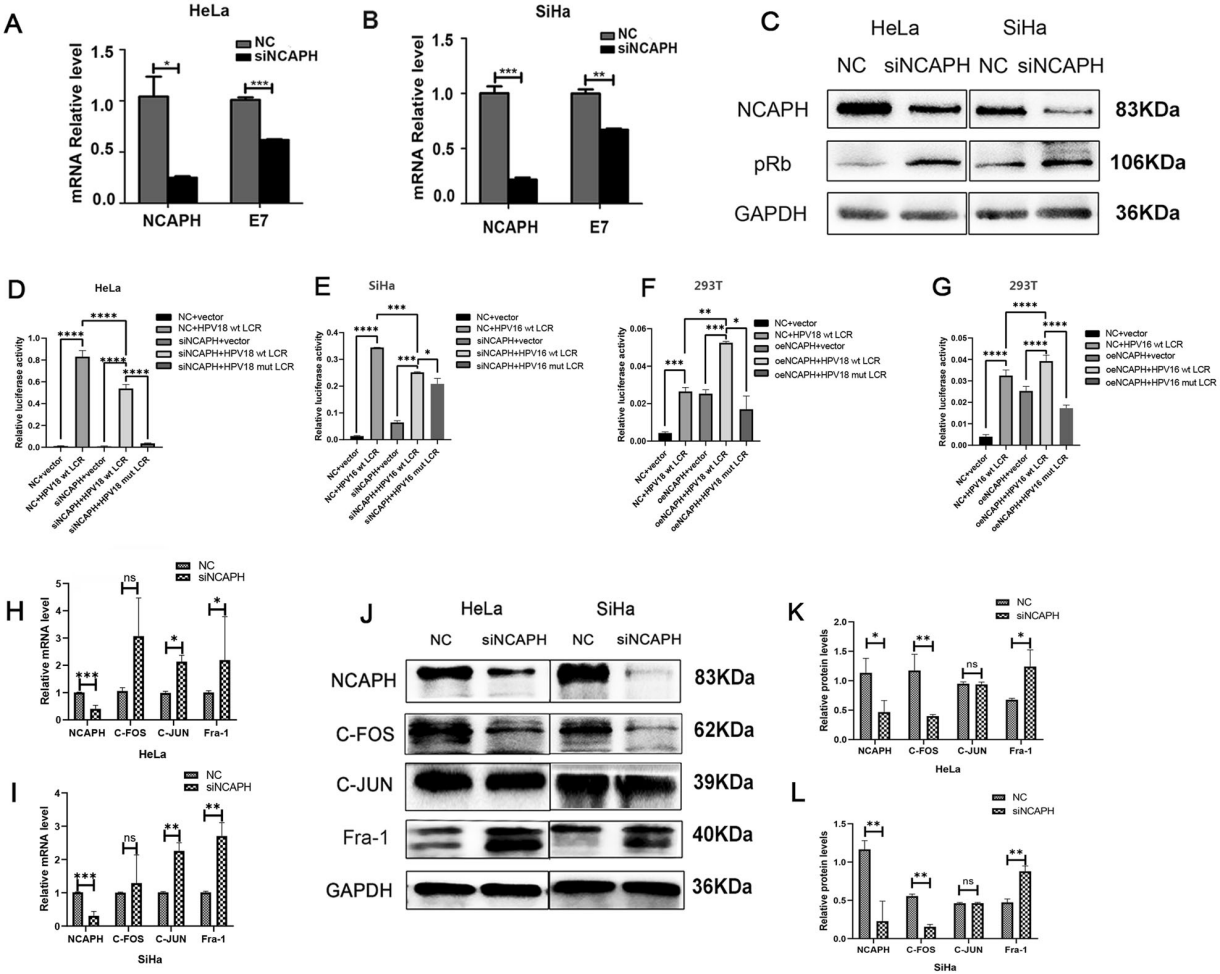
NCAPH trans-modulates the transcription of HPV E7 through the conversion of AP-1 components. **A**, **B** Real-time PCR showed that silencing NCAPH expression significantly decreased the mRNA level of E7 in HeLa and SiHa cells. **C** Western Blot analysis showed that knockdown of NCAPH increased the protein expression of pRb. **D**, **E** Luciferase assay showed the changes of fluorescence intensity after transfection in HeLa and SiHa cells. NC, negative control; siNCAPH, siRNA targeting NCAPH; vector, pGL3-Basic vector; HPV 18/16 wt LCR, pGL3 vector inserted with the wild type LCR; HPV 18/16 mut LCR, pGL3 vector inserted with the mutated LCR (mutation in the binding site of AP-1). **H**, **I** Luciferase assay showed the changes of fluorescence intensity after transfection in 293T cells. NC, mock vector; oeNCAPH, NCAPH over-expression plasmid. **F**, **G** Real-time PCR showed that mRNA levels of c-Fos, c-Jun, and Fra-1 were increased significantly after siNCAPH transfection in HeLa and SiHa cells. **H**–**L** Western Blot analysis showed that after silencing NCAPH expression in HeLa and SiHa cells, c-Fos protein expression was down-regulated, however Fra-1 protein levels were upregulated significantly. NC, negative control siRNA; siNCAPH, siRNA targeting NCAPH gene. Data are presented as mean ± SD (*n* = 3). * represents *p* < 0.05, ** represents *p* < 0.01, *** represents *p* < 0.001; ns not significant.

Intriguingly, when we knocked-down NCAPH gene expression in HeLa and SiHa cells, the mRNA levels of HPV E7 were reduced (Fig. 6A, B) and pRb protein level increased significantly (Fig. 6C–E). On the basis of our finding that E7 regulates the transcription of NCAPH, this result suggests that a positive feedback loop exists between HPV E7 and NCAPH.

Previous studies have shown that the LCR region of HPV E7 often acts as the binding sites for transcription factors and plays an essential role in the regulation of HPV gene expression. To elucidate whether NCAPH participates in the transcription regulation of E7 expression through the LCR region, luciferase assay was performed in HeLa, SiHa and 293T cells. Results showed that the fluorescence intensity increased greatly after the transfection of HPV 18 or 16 full-length LCR (Fig. 6D–G). It suggested that the viral promoter was active in all three kinds of cells, and HPV integration into host genome did not completely destroy the activity of HPV LCR in HeLa and SiHa. Moreover, when we knocked-down NCAPH in HeLa and SiHa transfected with the LCR, the fluorescence signal reduced significantly, and when we over-expressed NCAPH in 293T transfected with the LCR, the signal was further enhanced (Fig. 6D–G). The effects of NCAPH on the fluorescence intensity indicated that NCAPH was able to activate the transcription of the LCR. NCAPH might regulate E7 gene transcription by enhancing the activity of viral promoter in cervical cancer cells.

AP-1 is a key transcription factor that regulates E7 transcription by binding to LCR, and the conversion of AP-1 heterodimer regulates LCR activity significantly. Consistent with it, when we mutated the binding sites of AP-1 in HPV 16 and 18 LCR, the fluorescence intensity decreased greatly compared with the control group (Fig. 6 D–J). To unravel whether AP-1 participates in NCAPH-mediated LCR activation, we investigated the component changes of AP-1 transcription factor after interference with NCAPH in cervical cancer cells. Results showed that after interfering the expression of NCAPH, mRNA levels of c-Jun, c-Fos and Fra-1 were increased in HeLa and SiHa cells (Fig. 6H, I). At protein level, after silencing NCAPH expression in HeLa cells, c-Fos was down-regulated, while Fra-1 was up-regulated significantly; however, no significant change was observed for c-Jun level (Fig. 6J, K). Similar results were obtained in SiHa cells (Fig. 6J, L). These data indicated that silencing NCAPH could induce the conversion of AP-1 heterodimer from c-Fos/c-Jun to Fra-1/c-Jun, thus reduce its binding activity to HPV LCR and down-regulates HPV E7 transcription.

### Potential NCAPH involvement in the activation of the PI3K/AKT/SGK signaling pathway

**Fig. 7:**
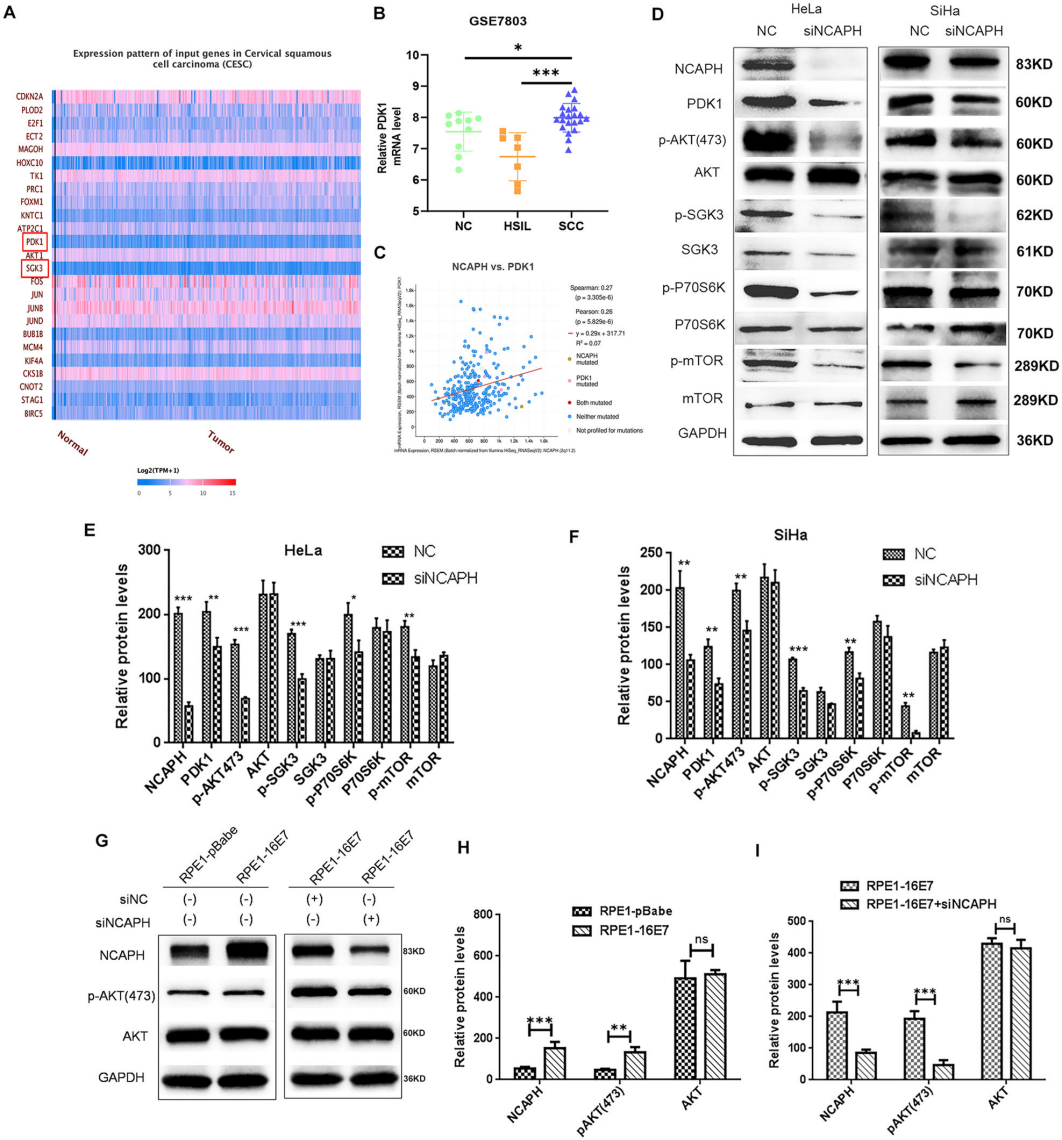
The involvement of E7 and NCAPH in the regulation of PI3K/AKT/SGK signaling pathway. **A** The UALCAN database showed that PDK1 was over-expressed in cervical cancer (tumor) compared with normal cervix (normal). **B** Analysis of GEO database (GSE7803) showed the NCAPH expression in normal cervix (NC), high-grade squamous intraepithelial lesion (HSIL) and cervical squamous cell cancer (SCC). **C** The cBioportal database revealed the co-expression of NCAPH and PDK1 in cervical cancer. **D**–**F** Western blot analysis showed the changes in the levels of key molecules of PI3K/AKT/SGK pathway after the knockdown of NCAPH expression in HeLa and SiHa cells. The relative protein level was analyzed. **G**–**I** Western blot analysis showed the protein levels of NCAPH, p-AKT(Ser473) and total AKT in RPE1-pBabe or RPE1-16E7 cells with or without transfection. The relative protein level was analyzed. NC, negative control siRNA; siNCAPH, siRNA targeting NCAPH gene. Data are presented as mean ± SD (*n* = 3). * represents *p* < 0.05, ** represents *p* < 0.01, *** represents *p* < 0.001, ns not significant.

**Fig. 8:**
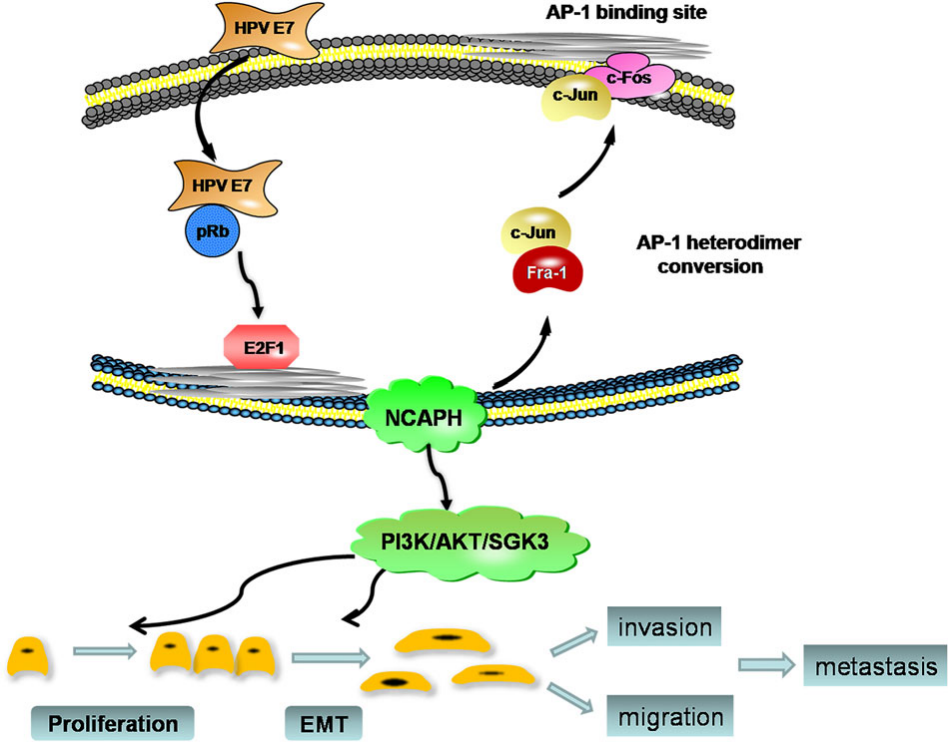
Proposed model for the role of NCAPH in carcinogenesis of HPV-induced cervical cancer. HPV E7 upregulates the transcription factor E2F1 by degrading pRb, and E2F1 binds with the promoter of NCAPH to activate its transcription. Reversely, NCAPH enhances the expression of E7 by promoting the transformation of AP-1 components from Fra-1/c-Jun to c-Fos/c-Jun. Overexpressed NCAPH promotes the development and progression of cervical cancer by the enhancement of proliferation, invasion and migration.

Analysis of UALCAN data showed that PDK1 and SGK3 were over-expressed in cervical cancer compared with normal cervix (Figs. 7A and 8). Analysis of GEO data (GSE7803) showed that the expression of PDK1 in cervical cancer was significantly higher than in normal cervix and HSIL separately (Fig. 7B). The data from cBioportal database revealed that NCAPH was significantly co-expressed with PDK1 in cervical cancer specimens (Fig. 7C).

To verify the above results, we examined whether NCAPH was involved in the regulation of the PI3K/AKT/SGK signaling pathway. Results showed that when the NCAPH expression was knocked down in HeLa and SiHa cells, the levels of AKT, SGK3, P70S6K and mTOR were unchanged, however the levels of PDK1, p-AKT(Ser473), p-SGK3(320), p-P^70S6K^ and p-mTOR were significantly decreased (Fig. 7D–F) (all *p* values < 0.05). These changes in the level of key signaling molecules suggest that NCAPH might play a key role in the activation of the PI3K/AKT/SGK signaling pathway.

Furthermore, we compared RPE1-pbabe cells with RPE1-16E7 cells. The results showed that the increase of NCAPH in E7 cells was accompanied by the increase of p-AKT(Ser473) protein level, however, the total Akt level was not changed. When we knocked down NCAPH expression in RPE1-16E7 cells, the protein level of p-AKT(Ser473) decreased significantly (Fig. 7G–I). These results suggest that E7 may be involved in activation of PI3K/AKT/SGK signaling pathway by activating Akt phosphorylation at ser473 site through NCAPH.

## Discussion

NCAPH is considered to be a protein involved in sister chromatid isolation during mitosis. At present, little is known about its role in human tumors. In this study, we have shown that silencing NCAPH expression reduced the proliferation, invasion, migration, and xenograft tumor formation ability of cervical cancer cells in vitro and in vivo. These factors imply that NCAPH could have an important role in promoting the progression of cervical cancer. These results are consistent with previous observations reported for colorectal cancer cells, lung cancer cells and breast cancer cells^18,19,24^. Therefore, NCAPH might be an essential oncogene not limited to cervical cancer more widely involved in the development and progression of various malignancies.

HPV E7 protein is a key virus-encoded carcinogenic factor. E7 can degrade pRb and release E2F, thereby regulating growth cycle and DNA repair, and inducing genomic instability. It has been proven that pRB, E2F1, and condensin II can localize to major satellite repeats at pericentromeres, forming a complex that ensures proper chromosome segregation in mitosis. These results suggest a possible association amongst E7, E2F1, and condensing proteins^25^. We found that E7 could enhance NCAPH gene expression by promoting the direct binding of E2F1 to the promoter of NCAPH gene. Considering the important role of NCAPH in the condensin complex, we speculate that although E7 up-regulated E2F1 and NCAPH expression, the degradation of pRb by E7 hinders the formation of pRb–E2F1–condensin complex, which leads to incorrect chromosome segregation, genomic instability, and tumorigenesis. However, the hypothesis needs to be further verified.

In recent years, researchers have made great progress in understanding the functions of E6 and E7. However, past researches mainly focused on how E7 regulates the expression of host genes, but how host genes transregulate the expression of E7 is still largely unknown^26^. Moreover, in order to resist the transformation of cells by the virus, most host proteins are proved to act as limiting factors to inhibit the replication and expression of HPV genes^26^. Different from previous studies, the mRNA level of E7 decreased significantly when we knocked down NCAPH in cervical cancer cells. It indicates that NCAPH can trans-modulate the transcription of E7. Considering the positive regulatory effect of E7 on NCAPH, this result suggests that a feedback loop exists between HPV E7 and NCAPH. However, the E7 protein is often considered to be a double-edged sword: the expression of E7 increases cell proliferation, while its overexpression induces cell death^3^. Similarly, the overexpression of NCAPH in cervical cancer cells induced severe cell death (data not shown). This suggests that the positive feedback loop of E7-NCAPH in cervical cancer cells is in equilibrium under the control of host cells. The overexpression of NCAPH might break the balance and upregulate the expression of E7, which eventually leads to cell death.

It is known that HPV genome is integrated into host chromosome in cervical cancer cells, however it is unclear whether NCAPH regulates E7 through host or viral promoters. Our data confirmed that the viral promoter was still active in cervical cancer cells which was consistent with previous reports^27^. Moreover, NCAPH could upregulate the activity of HPV LCR significantly. It indicated that NCAPH could regulate E7 through viral promoter. AP-1 is a key transcription factor responsible for the regulation of E7 promoter activity. It forms a heterodimer composed of Fos gene family (c-Fos, FosB, Fra-1, Fra-2) and Jun gene family (c-Jun, JunB, or JunD)^28,29^. During the malignant transformation of cervical cells, AP-1 complex transforms from Fra-1/c-Jun to c-Fos/c-Jun dimer, enhances the transcription of E7, and promotes the carcinogenesis process^30–32^. Consistent with it, we found that the mutation of AP-1 binding site in HPV LCR reduced the activity of LCR significantly. We speculated that NCAPH might regulate E7 transcription through AP-1. In line with this, we found that interference with NCAPH can significantly reduce the expression of c-Fos and increase the expression of Fra-1. It indicates that silencing NCAPH could induce the conversion of AP-1 heterodimers from c-Fos/c-Jun to Fra-1/c-Jun, reduce the activity of AP-1, and inhibit the transcription of E7. Moreover, the changes of AP-1 mRNA level after NCAPH interference were not completely consistent with the changes of protein level. It suggests that the regulation of NCAPH on c-Fos, c-Jun, and Fra-1gene expression may be complex, and its mechanism needs to be further clarified.

The PI3K/AKT pathway is an important signaling pathway, and there is clear evidence that amplification or mutation of the main components of PI3K/AKT pathway is common in cervical cancer and contributes to tumor progression^9,33–38^. HPV E6 and E7 could change intracellular and molecular events through PI3K/AKT signaling pathway, and promote malignant transformation of cervical cells^37^. However, the specific mechanisms are still unclear. In our study, we found that E7 was involved in the activation of PI3K/AKT signaling pathway partially through NCAPH. Silencing of NCAPH did not change the levels of AKT, SGK3, P70S6K, and mTOR, but significantly decreased their phosphorylation levels. The reduction in PDK1 expression when NCAPH expression is reduced suggests that, NCAPH could potentially activate the phosphorylation of AKT and SGK3 via upregulation of PDK1 expression. Bago et al. reported that AKT inhibitors increased the activation of SGK3 in the treatment of breast cancer, and SGK3 replaces AKT by phosphorylated TSC2, leading to the failure of AKT inhibitor treatment in some breast cancer patients^39^. Therefore, future therapy targeting NCAPH might have the potential to inhibit AKT and SGK simultaneously and exhibit better effects than AKT or SGK inhibitor treatment alone.

In cervical cancer, EMT plays an essential role in initiating cancer progression^40^. In the present study, silencing of NCAPH hindered the EMT process. The tight correlation between NCAPH and EMT was further confirmed in the xenograft tumor model suggesting NCAPH may promote EMT in cervical cancer cells. Furthermore, it is known that cells with a mesenchymal phenotype are prone to invading the lymphvascular cavities and enabling metastasis to distant sites^41^. The expression of NCAPH has consistently been associated with lymph node metastasis in our cervical cancer patients indicating that NCAPH might facilitate tumor invasion and metastasis by promoting EMT.

Recently, Zhai Y et al. revealed that NCAPH mRNA increased significantly from normal cervix to HSIL and from HSIL to ICSCC using high-density oligonucleotide microarrays. However, the results required further functional validation^42^. In our study, we assessed the expression of NCAPH in human cervical cancer tissues. We found NCAPH protein to be located both in cytoplasm and nucleus, a result that was consistent with a previous study that condensin I was present in the cytoplasm during interphase, and gained access to chromosomes only after breakdown of the nuclear envelope at the end of prophase^43^. Moreover, NCAPH expression in cervical cancer was significantly higher than those in normal cervix and HSIL. This result was in accordance with the observation in colon cancer patients^18^ and provides further evidence that NCAPH plays an important role in the initiation of cervical cancer. Although positive NCAPH expression was significantly associated with lymph node metastasis, its expression correlated with a better patient prognosis. This was opposite to the effect observed for non-small cell lung cancer^16^ while consistent with that in colon cancer^18^. Analysis of our patient cohort showed that the majority of the patients received adjuvant therapy because of high risk factors, such as lymph node metastasis (34/100) and deep invasion (74/100). Considering the vital role of NCAPH in cell proliferation, we hypothesized that tumors with positive NCAPH expression might have a higher proliferation rate, which could improve the effects of chemo- and/or radio-therapies and bring benefits to the patients’ prognosis. However, the result needs to be re-assessed in larger cohort of patients in the future.

In summary, we report that NCAPH is a novel oncogene that plays an important role in the initiation and progression of cervical cancer. The formation mechanism of feedback loop between HPV E7 and NCAPH deepens the molecular mechanism of HPV induced cervical cancer, and expands the mechanism of trans-regulation of host protein to HPV genes. In addition, NCAPH might be a novel multi-functional therapeutic target. Future personalized therapies targeting NCAPH is expected to induce robust antitumor responses not only reducing tumor growth ability, but also decreasing the expression of E7.

## Supplementary information

Supplementary Figure legends

Supplementary Fig. S1

Supplementary Fig. S2

Supplementary Fig. S3

Supplementary Table S1

Supplementary Table S2
