# Supplementary material for: HPV E7-mediated NCAPH ectopic expression regulates the carcinogenesis of cervical carcinoma via PI3K/AKT/SGK pathway
Source: Cell Death Dis. 2020 Dec 11;11(12):1049. doi: 10.1038/s41419-020-03244-9 (PMC7732835; doi:10.1038/s41419-020-03244-9)
Supplement: Supplementary file 1 — Supplementary Figure legends [file 41419_2020_3244_MOESM1_ESM.docx]

**Supplementary Figure legends:**

**Supplementary Figure. S1**. **The overexpression of NCAPH in cervical lesions and other solid tumors were confirmed by the data from the public database.**

**(A&B)** Data from GEO database (GSE 7803 and GSE138080); **(C&D)** Data from UALCAN database. Normal cervix (NC), high-grade squamous intraepithelial lesion (HSIL) and cervical squamous cell carcinoma (SCC). BLCA, Bladder urothelial carcinoma; BRCA, Breast invasive carcinoma; CESC, Cervical squamous carcinoma; CHOL, Cholangiocarcinoma; COAD, Colon adenocarcinoma; ESCA, Esophageal carcinoma; GBM, Glioblastoma multiforme; HNSC, Head and Neck squamous cell carcinoma; KICH：Kidney chromophobe; KIRC, Kidney renal clear cell carcinoma; KIRP, Kidney renal papillary cell carcinoma; LIHC, Liver hepatocellular carcinoma; LUAD, Lung adenocarcinoma; LUSC, Lung squamous cell carcinoma; PAAD, Pancreatic adenocarcinoma; PRAD, Prostate adenocarcinoma; PCPG, Pheochromocytoma and Paraganglioma; READ, Rectum adenocarcinoma; SARC, Sarcoma; SKCM, Skin cutaneous melanoma; THCA, Thyroid carcinoma; THYM, Thymoma; STAD, Stomach adenocarcinoma; UCEC, Uterine corpus endometrial carcinoma. * represents *p*<0.05, ** represents *p*<0.01, *** represents *p*<0.001, **** represents *p*<0.0001.

**Supplementary Figure. S2.** The cBioPortal database was used to analyze the genetic changes of NCAPH gene in cervical cancer studies. In 607 cases of cervical cancer, 1 case had NCAPH ampliﬁcation, 3 cases had missense mutation.

**Supplementary Figure. S 3**. The analysis of the cBioportal database revealed that NCAPH was significantly co-expressed with PCNA in 308 cases of cervical cancer patients indicating the essential role of NCAPH in promoting cell proliferation.
