# Supplementary figures and images for: HPV E7-mediated NCAPH ectopic expression regulates the carcinogenesis of cervical carcinoma via PI3K/AKT/SGK pathway
Source: Cell Death Dis. 2020 Dec 11;11(12):1049. doi: 10.1038/s41419-020-03244-9 (PMC7732835; doi:10.1038/s41419-020-03244-9)

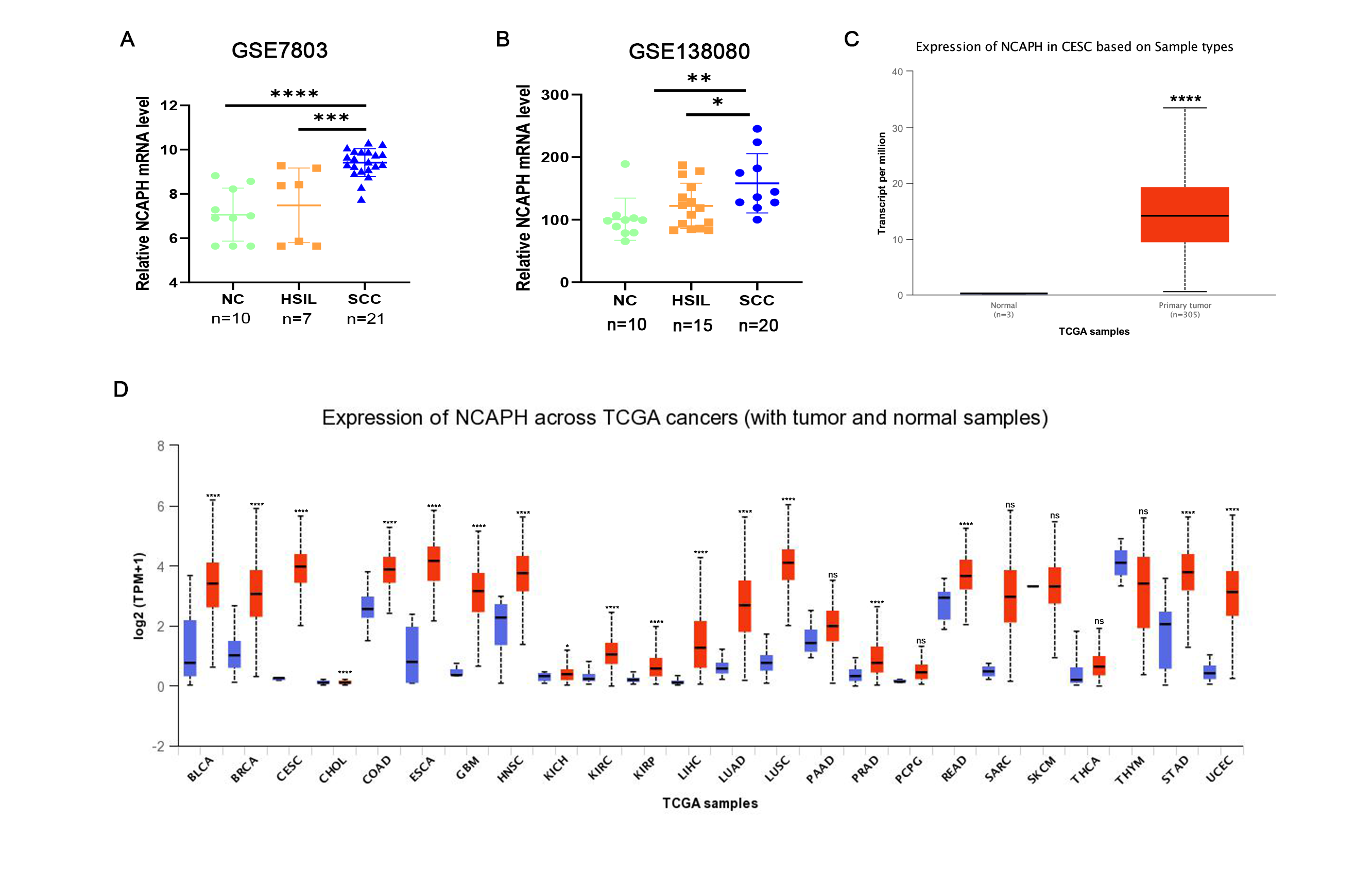

Supplement: Supplementary file 2 — Supplementary Fig. S1 [file 41419_2020_3244_MOESM2_ESM.tif]

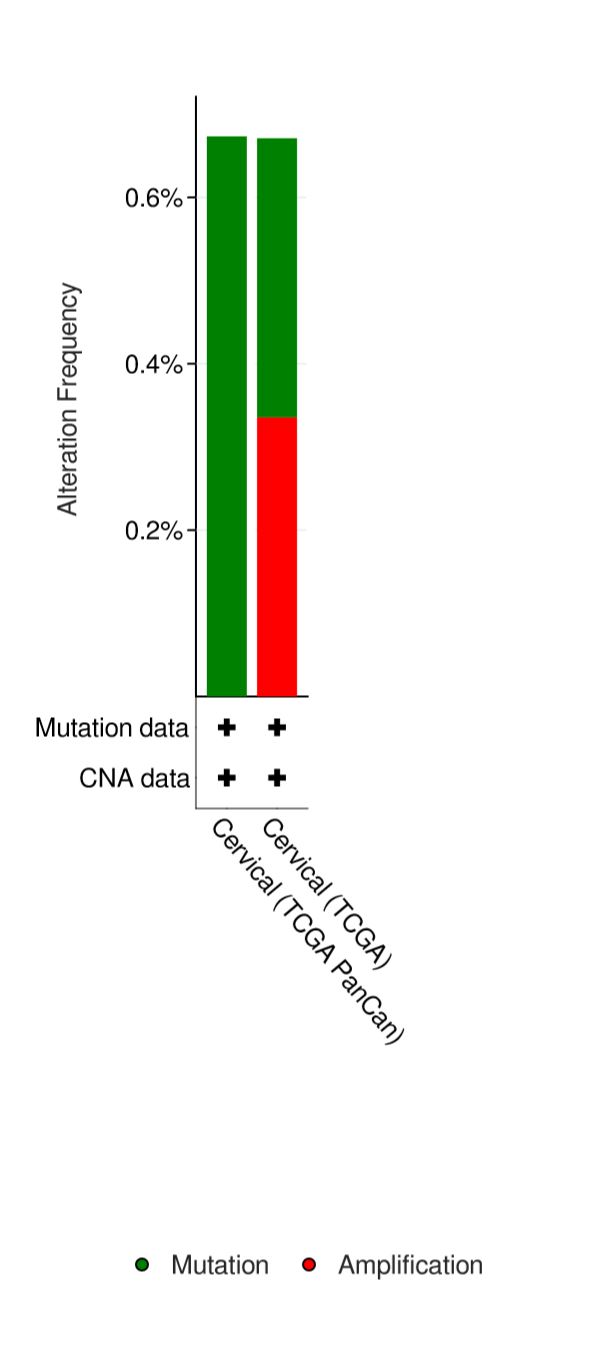

Supplement: Supplementary file 3 — Supplementary Fig. S2 [file 41419_2020_3244_MOESM3_ESM.jpg]

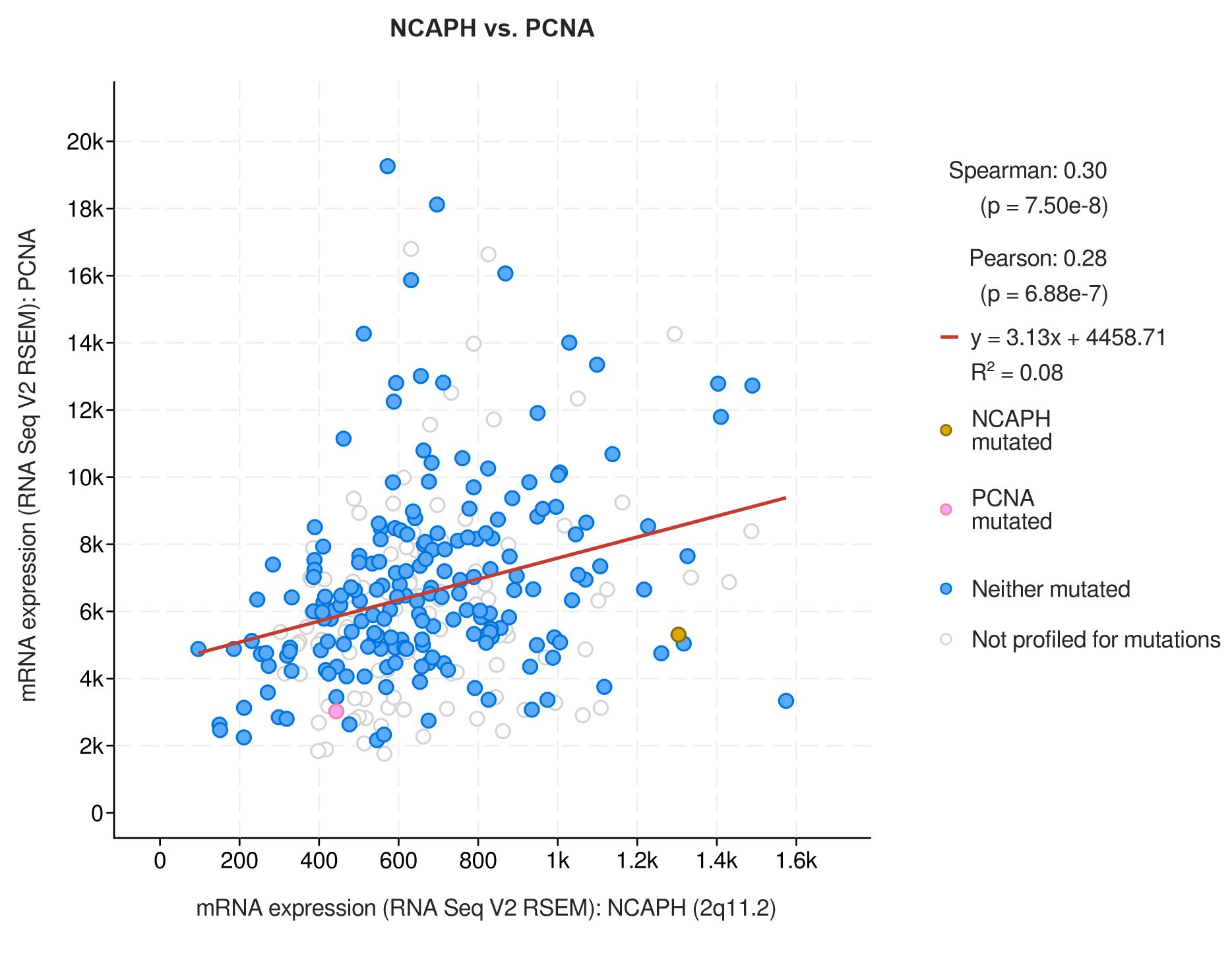

Supplement: Supplementary file 4 — Supplementary Fig. S3 [file 41419_2020_3244_MOESM4_ESM.jpg]
