# Supplementary material for: HPV E7-mediated NCAPH ectopic expression regulates the carcinogenesis of cervical carcinoma via PI3K/AKT/SGK pathway
Source: Cell Death Dis. 2020 Dec 11;11(12):1049. doi: 10.1038/s41419-020-03244-9 (PMC7732835; doi:10.1038/s41419-020-03244-9)
Supplement: Supplementary file 5 — Supplementary Table S1 [file 41419_2020_3244_MOESM5_ESM.docx]

**Supplementary Table S1**. The sequences of the primers and small interfering RNA (siRNA).

| **Gene names** | **The primer sequences** |
| --- | --- |
| HPV18 E7  HPV 16 E7  NCAPH  GAPDH | forward: 5’-TGTATTGCATTTAGAGCCCCAAA-3’  reverse: 5’-CTTCCTCTGAGTCGCTTAATTGC-3’  forward: 5’-AGTGTGACTCTACGCTTCGGTTG-3’  reverse: 5’-CTGAGAACAGATGGGGCACAC-3’  forward: 5’-ACAGTGCCTCCTCTCCTTCA-3’  reverse: 5’-CCGCTCCTTCTCATCGTCAT-3’  forward: 5’-GCACCGTCAAGGCTGAGAAC-3’  reverse: 5’-TGGTGAAGACGCCAGTGGA -3’ |
| **Gene names** | **The siRNA sequences** |
| HPV-16 E7  HPV-18 E7  NCAPH  E2F1  negative control | 5’-AGGAGGAUGAAAUAGAUGGTT-3’  5’-CUAGCACGAGCAAUUAAGCGA-3’  5’-GCCAGAGUUAGGUUGUGUATT-3’  5’-GUCACGCUAUGAGACCUCATT-3’  5’-UUCUCCGAACGUGUCACGUTT-3’ |
