# Supplementary material for: HPV E7-mediated NCAPH ectopic expression regulates the carcinogenesis of cervical carcinoma via PI3K/AKT/SGK pathway
Source: Cell Death Dis. 2020 Dec 11;11(12):1049. doi: 10.1038/s41419-020-03244-9 (PMC7732835; doi:10.1038/s41419-020-03244-9)
Supplement: Supplementary file 6 — Supplementary Table S2 [file 41419_2020_3244_MOESM6_ESM.docx]

**Supplementary Table S** **2.** Information of antibodies used in present study.

| Antibody | Catalog number | WB | IHC | Specificity | Company |
| --- | --- | --- | --- | --- | --- |
| NCAPH | 11515-1-AP | 1:1000 | 1:100 | Rabbit polyclonal | Proteintech, China |
| GAPDH | 10494-1-AP | 1:5000 | 1:100 | Rabbit polyclonal | Proteintech, China |
| Vimentin | 10366-1-AP | 1:2000 | 1:1500 | Rabbit polyclonal | Proteintech, China |
| ZO-1 | 21773-1-AP | 1:1000 | 1:100 | Rabbit polyclonal | Proteintech, China |
| Snail | 13099-1-AP | 1:1000 |  | Rabbit polyclonal | Proteintech, China |
| pRb | sc-102 | 1:1000 | 1:100 | Mouse monoclonal | Santa Cruz, USA |
| C-FOS | sc-166940 | 1:1000 | 1:100 | Mouse monoclonal | Santa Cruz, USA |
| C-JUN | sc-74543- | 1:1000 | 1:100 | Mouse monoclonal | Santa Cruz, USA |
| Fra-1 | sc-376148 | 1:1000 | 1:100 | Mouse monoclonal | Santa Cruz, USA |
| E2F1 | sc-251 | 1:1000 | 1:100 | Mouse monoclonal | Santa Cruz, USA |
| PDK1 | 5662S | 1:1000 |  | Rabbit monoclonal | CST, USA |
| AKT | 4685S | 1:1000 | 1:200 | Rabbit monoclonal | CST, USA |
| P-AKT | 4060S | 1:2000 | 1:100 | Rabbit monoclonal | CST, USA |
| SGK3 | 8156S | 1:1000 |  | Rabbit monoclonal | CST, USA |
| P-SGK3 | 5642S | 1:1000 |  | Rabbit monoclonal | CST, USA |
| P70S6K | 2708S | 1:1000 |  | Rabbit monoclonal | CST, USA |
| p-P70S6K | 9204S | 1:1000 |  | Rabbit monoclonal | CST, USA |
| mTOR | 2983S | 1:1000 |  | Rabbit monoclonal | CST, USA |
| p-mTOR | 5536S | 1:1000 |  | Rabbit monoclonal | CST, USA |
